# Predicting joint loading in Asian overweight and obese females with flexible flatfoot: a regression analysis of anthropometric parameters and gait dynamics

**DOI:** 10.1007/s11517-025-03378-y

**Published:** 2025-06-09

**Authors:** Linjuan Wei, Guoxin Zhang, Tony Lin-Wei Chen, Yan Wang, Yinghu Peng, Ming Zhang

**Affiliations:** 1 Department of Biomedical Engineering, Faculty of Engineering, The Hong Kong Polytechnic University, Hong Kong, 999077 China; 2 Research Institute for Sports Science and Technology, The Hong Kong Polytechnic University, Hong Kong, 999077 China; 3 The Hong Kong Polytechnic University Shenzhen Research Institute, Shenzhen, 518057 China; 4 Institutes of Advanced Technology, CAS Key Laboratory of Human-Machine Intelligence-Synergy Systems, Chinese Academy of Sciences, Shenzhen, 518055 China

**Keywords:** Anthropometrics, Flexible flatfoot, Kinetics, Regression analysis

## Abstract

Current methods for obtaining accurate joint loading data lack simplicity, efficiency, and cost-effectiveness. This study aims to generate joint loading prediction models using anthropometric parameters and walking speed in overweight or obese females with flexible flatfoot. Sixteen participants’ motion capture data from walking trails and anthropometric parameters were collected. The lower limb joint contact forces and the walking speed were calculated via a musculoskeletal model. Regression analysis was used to generate the prediction model. The second peak of knee joint contact force revealed a strong negative correlation with hip circumference and a weak positive correlation with age (*p* < 0.001 and adjusted *R*^2^ = 0.720). The peak ankle joint contact force exhibited a strong positive correlation with walking speed while strong negative correlations with waist circumference and lower limb length (*p* < 0.001 and adjusted *R*^2^ = 0.782). The first peak of vertical GRF displayed a medium negative correlation with walking speed (*p* < 0.001 and adjusted *R*^2^ = 0.750). Anthropometric parameters and walking speed are effective predictors of joint loading. This rapid, low-cost estimation method can be applied to areas such as flexible flatfoot that require assessment of joint stress, thereby saving costs and time.

**Graphical abstract:**

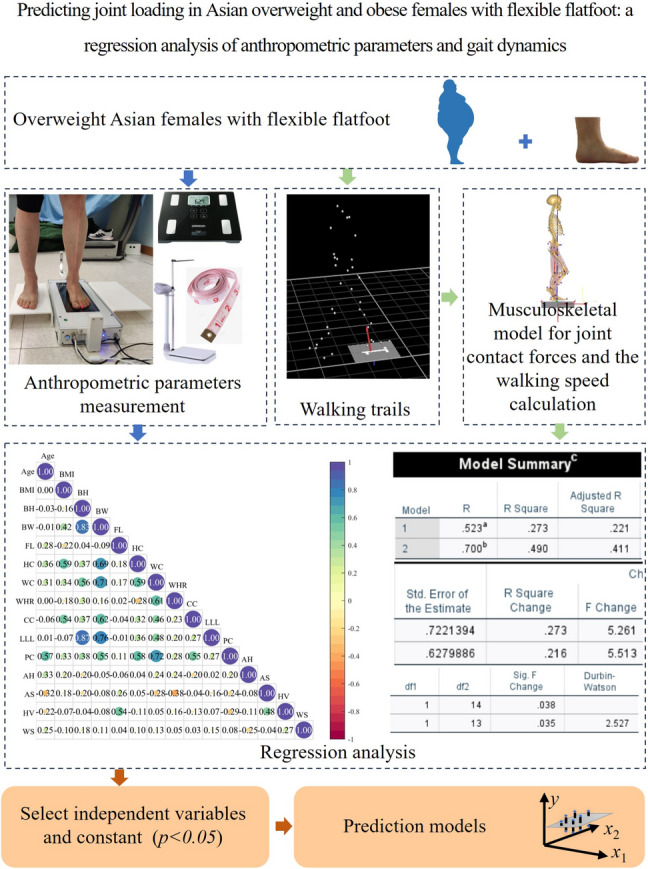

## Introduction

Overweight and obesity are global health issues that are associated with greater risks of lower limb musculoskeletal disorders [1]. Studies indicate that around 27 to 75% of overweight and obese individuals are affected by flatfoot [2–4], a foot deformity that is characterized by collapsed foot arch and subsequent changes in the lower limb mechanical alignment. Females are more prone to developing flatfoot than males [5], mainly due to the hormonal influences and anatomical features [6–9]. Given the kinetic coupling of adjacent body segments during locomotion, flatfoot can disrupt the kinetic chain and increase force distribution on the lower limb joints [10]. Obesity will further worsen the mechanical conduction and force distribution of flatfoot patients, as the increased weight-bearing burden on the articular surface [11] and dysregulation of joint force caused by fat-induced muscle weakness. The coexistence of obesity and flatfoot will boost the risk of sequential joint pathologies, such as knee osteoarthritis and ankle instability [6, 7, 12–14], since excessive joint loading contributes to articular wear and degeneration. Some intervention measures have been widely used, such as orthopedic insoles or orthopedic surgery, to reduce the negative impact of flatfoot on the musculoskeletal system. The first step in diagnosing whether and how to intervene in flatfoot in obese women is to assess the joint loading accurately.

Previous studies have employed various methodologies, such as implanted stress gauge [15–17] and musculoskeletal modeling [18], to quantify lower limb joint forces. Despite the existing findings, these approaches possess many limitations. Implanted gauge is an invasive method that can provide precise measurements of joint loading. Yet, it inherits the risk of infection and tissue damage, as well as presents ethical dilemmas, particularly when the investigation involves fragile groups [19]. On a frequent basis, invasive methods also restrict natural joint movement, potentially leading to a gait pattern that underrepresents the actual loading profile [20]. The modeling method uses rigid-body simulation and inverse dynamics to derive joint forces from body segment kinematics and ground reaction forces [21]. Kinematic data is usually acquired through motion-tracking techniques, such as marker-based motion capture analysis and wearable inertial measurement sensors [15–17]. Aside from requiring a time-consuming procedure for setup, the modeling methods usually require expert knowledge to achieve simulation convergence. In addition, the costly computational resource for successful kinetics calculation is not generally accessible in a clinician’s practice. Therefore, there is an urgent need for an effortless system that can accurately predict joint loadings based on simple measures, such as anthropometries and walking speed.

Machine learning (ML) has recently emerged as an advanced prediction model that revolutionizes the paradigm of biomechanical research [22], including the route of analyzing the human musculoskeletal system [23], such as the prediction of continuous joint kinetics based on measures of muscle electrophysiology [24] and inertial tracking of the body segments [25, 26]. However, these advancements are achieved using complex laboratory settings and are derived from asymptomatic individuals. The predictability of joint force in groups of obesity and flatfoot by ML models is unclear. Quantifying the lower limb joint forces can provide evidence to indicate the stress level of the joints and inform proactive measures for preventing injuries.

In this study, we aimed to develop an ML model, a stepwise multiple linear regression model, to predict joint loading among obese females with flexible flatfoot based on anthropometric metrics and walking speed. Flatfoot is classified into two primary types: rigid flatfoot and flexible flatfoot. Flexible flatfoot, the most common variant, is characterized by a reducible medial longitudinal arch that restores during non-weight-bearing conditions [27, 28]. The anthropometric data was manually measured under the supervision of a physical therapist, and the joint loading was derived from gait analysis. Our methodology aims to streamline joint loading estimation without compromising precision, focusing specifically on females with obesity and flexible flatfoot. We hypothesized that anthropometric parameters and walking speed would be reliable predictors for joint loading.

## Materials and methods

### Study cohort

Sixteen participants were recruited in this study. The inclusion criteria were as follows: (1) females aged 18–40 years; (2) BMI ≥ 23 kg/m2 [29]; (3) navicular drop ≥ 10 mm on one or both sides of feet; and (4) the tiptoe test showing a reconstructed medial arch when the subjects switched from a standing position to a low weight bearing position (sitting) [28]. The exclusion criteria included (1) foot deformities other than flexible flatfoot, damage or treatment for flexible flatfoot within the past 6 months, such as congenital clubfoot, amputation of the foot, and severe diabetic foot; (2) potential diseases that can influence walking and running ability; and (3) other diseases that were not suitable for walking experiments, such as high blood pressure. Participants were fully informed of this study and signed the informed consent. This study was performed in compliance with the declaration of Helsinki and approved by the institutional review board (Reference No: HSEARS20220318002).

### Experiment protocol

**Fig. 1:**
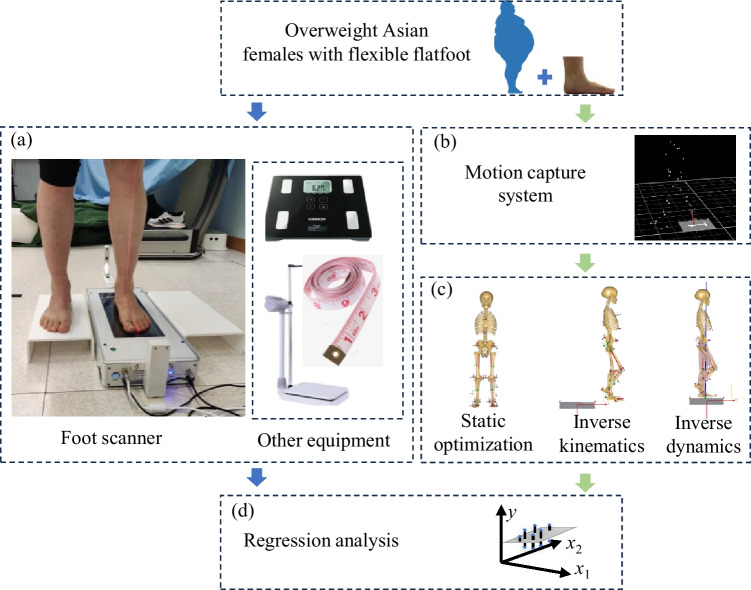
The flow chart of this study. **a** Anthropometric parameters measurement; **b** walking experiments; **c** calculation of joint forces and the walking speed; **d** regression model development

A commercial foot scanner (IFOOT-USOL, Shenzhen, China) [30] was used in this study to measure the heel alignment level, shank alignment level, and hallux valgus level at a standing position of 50/50 body weight distribution over the two feet (Fig. 1a). After the foot assessment, the body height, body weight, and circumferences of the breast, waist, pelvis, and hip were measured according to the landmarks of the human body [31–35] (Fig. 1a). The foot assessment and the measurement of anthropometric parameters were conducted under the supervision of a physical therapist. The anthropometric parameters collected in this study were body height, body weight, BMI, foot length, lower limb length, heel alignment level, shank alignment level, hallux valgus level, hip circumference, pelvis circumference, waist circumference, chest circumference, and waist-to-hip ratio.

A motion capture system with eight infrared cameras (Vicon Motion System Ltd., Oxford, UK) and six force platforms (OR6, AMTI, Watertown, USA) were used to obtain body trajectories and ground reaction force (GRF) (Fig. 1b). To enable motion capture, a total of 34 reflective markers were placed on anatomic landmarks [36]: the 2nd and 5 th metatarsal heads, the apex of the medial and lateral malleolus, shanks, lateral and medial femoral epicondyles, thigh, left and right greater trochanters, left and right anterior superior iliac spines, left and right iliac crests, the xiphoid process of the sternum, jugular notch, the 7 th cervical vertebrae, and the 7 th thoracic vertebra. A five-minute warm-up adaptation was conducted before the walking experiment. All participants walked at their individual’s preferred speed along a 10 m walkway. A static calibration trial was collected prior to the walking experiment to scale the musculoskeletal model. For each participant, five trials were collected in which the entire left stance phase was captured within the force plate. The marker trajectories and GRF were collected synchronously at a frequency of 250 Hz and 2000 Hz, respectively.

### Calculation of joint forces and the walking speed

Marker trajectories and GRF data were processed using a 4 th-order bidirectional Butterworth low-pass filter with cutoff frequencies set at 6 Hz and 50 Hz, respectively. These filtered marker trajectories and GRF data were then input into the musculoskeletal modeling software AnyBody (Version 7.4; AnyBody Technology, Aalborg, Denmark) to compute joint forces and walking speed through biomechanical simulations. We used the lower limb musculoskeletal multibody model that was well established in previous studies [37] and accommodated the fat percentage setting to females. The model's computational approach consisted of three steps: static optimization, inverse kinematics, and inverse dynamics [36]. In the static optimization stage, the bone geometry was scaled, and the locations of virtual markers in the model were determined using static trial data. Walking speed was calculated through inverse kinematics of the walking trials. Inverse dynamics were then executed to compute the joint contact forces (Fig. 1c). The joint force was normalized by the individual’s body weight.

### Regression model development

**Fig. 2:**
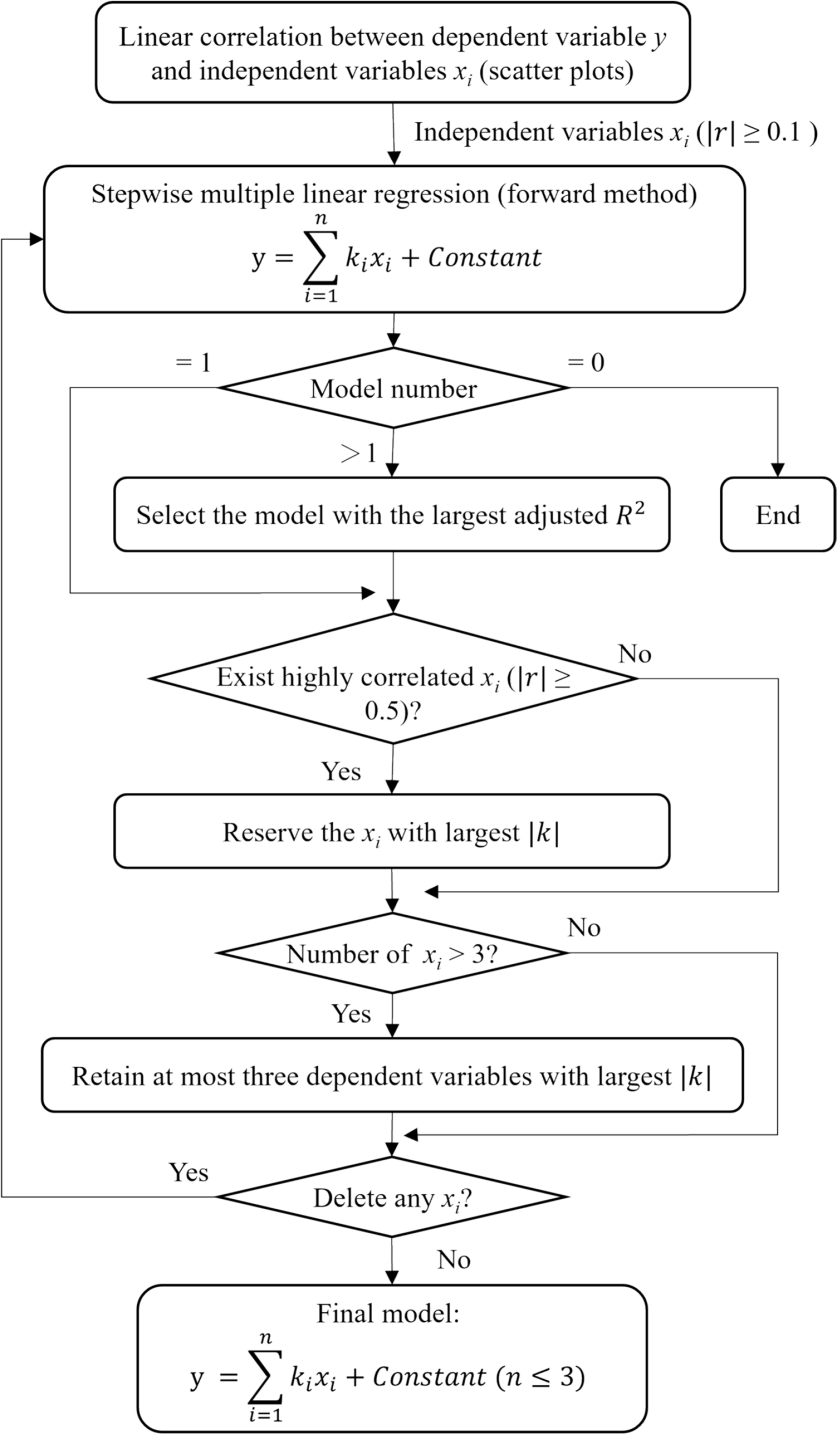
The framework flow for model development

The stepwise multiple regression model was built using commercial statistical software (SPSS 27; IBM, Armonk, NY): $y = {\sum }_{i=1}^{n}{k}_{i}{x}_{i}+ \text{constant}$ (Fig. 2). Where, $y$ is the dependent variable, ${x}_{i}$ and ${k}_{i}$ are the $i$-th independent variables and corresponding coefficient, respectively. Dependent variables of the regression model were the first and second peak values of vertical GRF and contact forces at the hip, knee, patellofemoral, and ankle joints. The anthropometric parameters and walking speed were used as the independent variables in this study. Normalization was first applied to all continuous variables. Multicollinearity among the independent variables was examined by Pearson correlation analysis [38]. For each dependent variable, its linear correlation with the independent variables was determined using scatter plots [39]. In linear regression analysis, the sample size is at least five times the independent variables [40]. The number of participants in this study is 16, so each regression model in this study can retain at most three dependent variables, and the ones with large absolute values of coefficients are retained first. Independent variables that had a linear correlation efficient ($\left|r\right|$ ≥ 0.1) with the dependent variable were analyzed by stepwise multiple linear regression (forward method) with a significance level of *p* < 0.05. When more than one model was generated, the one with the largest adjusted *R*^2^ was selected. If there exist highly correlated independent variables ($\left|r\right|$ ≥ 0.5) [38, 39, 41] in the generated model, the independent variable with large absolute values of coefficient was reserved to accurately interpret the influence of independent variables on the corresponding dependent variable.

## Results

### Pearson correlation analysis among independent variables

**Fig. 3:**
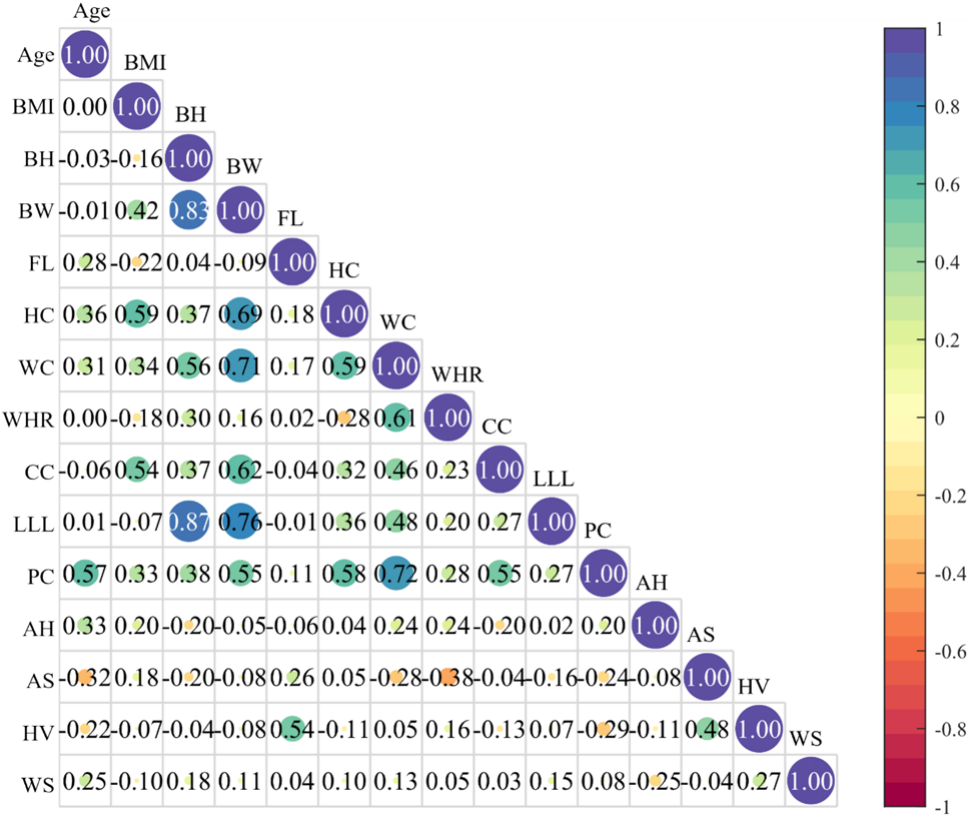
The results of the Pearson correlation analysis for independent variables. Abbreviations: AH = alignment level of the heel; AS = alignment level of the shank; BH = body height; BW = body weight; CC = chest circumference; FL = foot length; HC = hip circumference; HV = hallux valgus level; LLL = lower limb length; PC = pelvis circumference; WC = waist circumference; WHR = waist-to-hip ratio; WS = walking speed

The detailed results of anthropometric measures and kinematic parameters are displayed in Appendix Table 3 and Appendix Table 4. All the continuous independent variables showed normal distributions. The results of the Pearson correlation analysis for independent variables in this study are shown in Fig. 3. Age and waist-to-hip ratio were correlated with the lowest number of independent variables, which were pelvis circumference and waist circumference. In contrast, body weight was significantly correlated with seven other independent variables. Waist and pelvis circumference have strong correlations with five other independent variables, while chest circumference, lower limb length, and foot length have strong correlations with three other independent variables. Body height and hip circumference have strong correlations with four other independent variables, respectively. Walking speed was not correlated with any anthropometric parameters.

### Independent variables for stepwise multiple linear regression

**Fig. 4:**
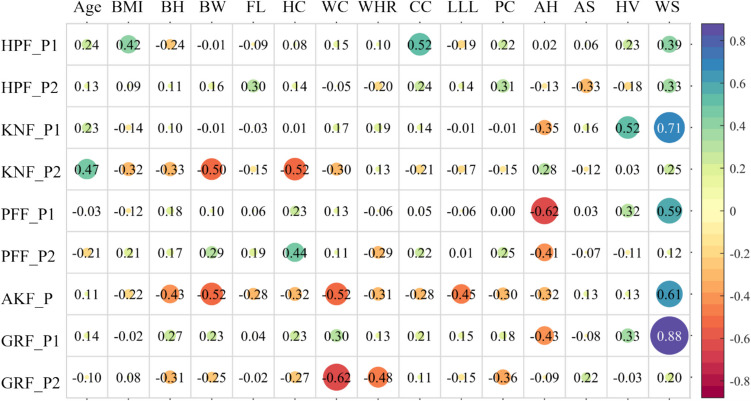
The results of the Pearson correlation analysis for independent variables and dependent factors. Abbreviations: AH = alignment level of the heel; AKF_P = the peak value of ankle joint contact force; AS = alignment level of the shank; BH = body height; BW = body weight; CC = chest circumference; FL = foot length; HC = hip circumference; HPF_P1 = the first peak of hip joint contact force; HPF_P2 = the second peak of hip joint contact force; HV = hallux valgus level; KNF_P1 = the first peak of knee joint contact force; KNF_P2 = the second peak of knee joint contact force; LLL = lower limb length; PC = pelvis circumference; VGR_P1 = the first peak of vertical ground reaction force; VGR_P2 = the second peak of vertical ground reaction force; WC = waist circumference; WHR = waist-to-hip ratio; WS = walking speed

Pearson correlation analysis among independent variables is displayed in Fig. 4. Table 1 summarizes the independent variables that were included in stepwise multiple linear regression based on the results of the Pearson correlation analysis. Table 2 presents the final prediction models derived from stepwise multiple linear regression, including regression coefficients, overall model significance (*p*), and adjusted *R*^2^ values quantifying the proportion of variance explained. For example, the model predicting the first peak of ankle joint contact force (AKF first peak = 11.045 + 2.027 × WS − 5.849 × WC − 5.110 × LLL) demonstrated a statistically significant association (*p* = 0.001), accounting for 78.2% of the variance in the AKF first peak (adjusted *R*^2^ = 0.782). The positive coefficient for walking speed (WS: 2.027) suggests that faster walking speeds were associated with an increase in the AKF first peak. Conversely, the negative coefficients for waist circumference (WC: − 5.849) and lower-limb length (LLL: − 5.110) indicate that smaller waist circumferences and shorter lower-limb lengths were linked to a reduction in the AKF first peak. There were no significant predictors of the second peak of the hip contact force and patellofemoral contact force. Walking speed was the only regressor for the first peak of the knee joint contact force and vertical GRF. Two regressors were included in the models to predict the peak ankle contact force and the second peak of the knee contact force, patellofemoral contact force, and vertical GRF. Three regressors, the chest circumference, body height, and walking speed, were required to predict the first peak of the hip contact force based on the analysis outcomes.

**Table 1:** The Pearson correlation between dependent variable and the chosen independent variables among each regression model

Dependent variables	Independent variables
HPF’s first peak	Chest circumference (*r* = 0.52), body height (*r* = − 0.24), walking speed (*r* = 0.39)
KNF’s first peak	Walking speed (*r* = 0.71)
KNF second peak	Hip circumference (*r* = − 0.52), age (*r* = 0.47)
PFF’s first peak	Heel alignment (*r* = − 0.62), walking speed (*r* = 0.59)
AKF’s peak	Waist circumference (*r* = − 0.52), lower limb length (*r* = − 0.45)
GRF’s first peak	Walking speed (*r* = 0.88)
GRF’s second peak	Waist circumference (*r* = − 0.62), chest circumference (*r* = 0.11)

Abbreviations: *AKF*, ankle joint contact force; *GRF*, ground reaction force; *HPF*, hip joint contact force; *KNF*, knee joint contact force; *PFF*, patella-femur joint contact force; *WS*, walking speed

**Table 2:** Detailed information of the generated prediction models

Prediction models	*p*	Adjusted *R*^2^
HPF first peak = 3.783 + 10.506 × CC – 7.311 × BH + 1.670 × WS	0.001	0.642
KNF first peak = − 0.145 + 2.074 × WS	0.002	0.474
KNF second peak = 13.033 − 12.274 × HC + 0.074 × age	< 0.001	0.720
PFF first peak = 0.134–0.468 × AH + 1.499 × WS	0.004	0.515
AKF peak = 11.045 + 2.027 × WS − 5.849 × WC − 5.110 × LLL	< 0.001	0.782
VGRF first peak = 0.646 − 0.357 × WS	< 0.001	0.750
VGRF second peak = 1.340 − 0.751 × WC + 0.397 × CC	0.003	0.520

Abbreviation: *AH*, alignment level of the heel; *AKF*, ankle joint contact force; *BH*, body height; CC, chest circumference; *HC*, hip circumference; *HPF*, hip joint contact force; *KNF*, knee joint contact force; *LLL*, lower limb length; *PFF*, patella-femur joint contact force; *VGRF*, vertical ground reaction force; *WC*, waist circumference; *WS*, walking speed

## Discussion

Overweight and obesity impair the functionality of the musculoskeletal system, particularly in females with flatfoot. Kinetic data can reflect joint loading and indicate the potential mechanism behind joint degeneration and arthritis [42]. Understanding joint kinetics is essential for conservative treatments of lower limb deformities, such as custom insole interventions for flexible flatfoot. Our study showed that ML models can be used to predict joint loading by acquiring anthropometric metrics and walking speed. These ML models are based on regression analyses of the joint contact forces.

Although seven regression models were generated that represent the specific predictors that could statistically significantly predict the outcomes, four of them were reported with adjusted *R*^2^ below 0.7. According to a previous study about the prediction of the kinetics [43], the adjusted *R*^2^ below 0.7 indicates a powerless prediction. Three of those linear models showed good ability for the prediction, including the second peak value of the knee joint force (*p* < 0.001, adjusted *R*^2^ = 0.720), the peak value of the ankle joint force (*p* < 0.001, adjusted *R*^2^ = 0.782), and the first peak value of the vertical ground reaction force (*p* < 0.001, adjusted *R*^2^ = 0.750).

The knee contact force is an important indicator of the prognosis of knee osteoarthritis on a clinical basis [44]. Our findings revealed that the second peak value of knee joint force exhibited a strong negative correlation with hip circumference and a weak positive correlation with age. Although a larger hip circumference is often associated with greater body weight, which can lead to increased knee joint contact forces [45], among individuals with the same body weight, a larger hip circumference may signify stronger lower limb and core muscle strength [46]. Stronger muscles could better absorb and distribute the forces generated during walking, thus reducing the peak forces transmitted to the knee joint [46, 47]. Conversely, older adults may experience higher knee joint forces compared to younger individuals with the same body weight and hip circumference, due to age-related declines in muscle mass and strength [48]. Collectively, these results highlight that, under equivalent body weight conditions, smaller hip circumference (a potential marker of reduced muscle strength) and advanced age (a proxy for muscle deterioration) are critical risk factors for elevated knee joint forces and, by extension, knee osteoarthritis.

This study demonstrated that the peak ankle joint force has a strong positive correlation with walking speed, while it has strong negative correlations with waist circumference and lower limb length. An increased walking speed is likely achieved through increased force output of the calf muscles and rectus femoris for forward propulsion [49, 50]. Consequently, an increased calf muscle force contributes to higher ankle contact force [51]. In general, a larger waist circumference is likely caused by fat accumulation and is related to the anterior and inferior displacement of the mass center [52], which can incur a higher plantarflexion force on the ankle joint to counterbalance the postural deviation [53]. Surprisingly, our research indicates a negative correlation between ankle joint contact force and waist circumference. A plausible explanation for this finding is that the joint force is normalized by body weight in this study. An increase in waist circumference is generally indicative of greater fat accumulation around the abdominal area. When body weight is constant, this suggests a redistribution of mass, possibly with less muscle mass in the lower body. Take the eighth and fifteenth participants as examples (Appendix Tables 3 and 4). These two participants have similar body height and body weight (also similar lower limb length and hip circumference). However, the eighth participant has a much bigger waist circumference (0.84 vs. 0.772 m), which means the eighth participant has more weight (fat) distributed in the abdomen and less weight (muscle) distributed in the lower extremities compared to the fifteenth participant. Since muscle strength is a key factor in generating force, a decrease in lower body muscle mass could lead to reduced peak force production at the ankle joint [54]. Additionally, individuals with longer limbs experience an increase in the moment arm length, which can decrease the contractile force required from the soleus muscle, thereby optimizing the plantar angle of the ankle during the propulsion phase of movement [55, 56]. Therefore, a diminished peak ankle joint force implies that longer limbs might necessitate less muscle force to produce the same amount of torque around the ankle joint.

The initial peak of the vertical ground reaction force exhibited a slight negative correlation with walking speed. Previous research indicated that for healthy individuals, the first peak value of the vertical joint contact force escalates with increasing speed [57]. The discrepancy in these findings could be attributed to the fact that our study's increment in walking speed may only occur during the propulsion phase [49]. This inference is inferred from the association between the walking speed and both the peak value of the ankle joint contact force and the initial peak of the vertical ground reaction force. Further investigation is needed to determine the precise reason for the negative relationship between the first peak value of the vertical joint contact force and the walking speed.

Several limitations existed in this study. This study used a small sample size to estimate the regression coefficients, which may have biases, although this avoids the disadvantage of overfitting [58]. We try to reduce the bias of parameters by reducing the number of explanatory variables [40]. Still, the limited sample size also limits the number of explanatory variables we can use, which compromises the accuracy of the model and may cause the miss of important explanatory variables. This study only involved overweight or obese Asian females with flexible flatfoot, which means the findings in this study may not be generalizable to other cohorts with different demographical backgrounds and fitness levels [59], such as females with normal feet, as the flatfoot changes the mechanical alignment and impair normal gait patterns [8]. Future studies may consider using a bigger sample size with different demographic backgrounds and fitness levels to improve the estimation precision and generalizability of the proposed method of joint loading prediction based on anthropometric parameters, walking speed, and regression model.

## Conclusion

In this study, we employed anthropometric parameters and walking speed to estimate joint contact forces and GRFs for overweight and obese females with FFF through regression analysis. The second peak of knee joint contact force exhibited a strong negative correlation with hip circumference and a weak positive correlation with age. The peak ankle joint contact force showed a strong positive correlation with walking speed while strong negative correlations with waist circumference and lower limb length. The first peak of vertical GRF displayed a medium negative correlation with walking speed. Overall, this study indicated that anthropometric parameters and walking speed can be utilized to estimate joint loading using a regression model. This rapid assessment method, which is not based on expensive laboratory instruments, can be applied to areas such as flexible flatfoot screening and diagnosis and treatment that require assessment of joint stress, thereby saving considerable costs and time for relevant personnel and institutions. Future studies may consider applying the method of predicting joint force based on anthropometric parameters, walking speed, and regression models to more clinical areas and improving the generalization ability of the prediction model by increasing the sample size.

## Appendix

Tables 3 and 4.

**Table 3:** The results of anthropometric measurements of the participants

No	*A* (years)	*B* (kg/m^2^)	*C* (m)	*D* (kg)	*E* (m)	*F* (m)	*G* (m)	*H* (1)	*I* (m)	*J* (m)	*K* (m)	*L*	*M*	*N*	WS (m/s)
1	37	23.94	1.595	60.9	0.2397	1.04	0.88	0.8462	0.905	0.828	0.958	2	2	1	1.52
2	34	27.78	1.495	62.1	0.2248	1.013	0.85	0.8391	1.041	0.785	0.975	2	1	0	1.39
3	23	25.11	1.625	66.3	0.2434	1	0.795	0.7950	0.94	0.834	0.94	2	2	0	1.17
4	31	23.09	1.585	58	0.2269	0.95	0.83	0.8737	0.861	0.78	0.895	1	0	0	1.71
5	21	23.95	1.613	62.3	0.2302	0.97	0.85	0.8763	0.95	0.825	0.925	1	1	0	1.12
6	34	25.01	1.507	56.8	0.2376	0.97	0.815	0.8402	0.9	0.78	0.94	3	1	0	1.57
7	39	27.05	1.637	72.5	0.2401	1.108	0.93	0.8394	0.93	0.86	0.99	3	1	0	1.18
8	22	24.02	1.6	61.5	0.2494	0.96	0.84	0.8750	0.885	0.87	0.89	3	0	0	0.93
9	22	26.63	1.635	71.2	0.2446	1.1003	0.88	0.7998	0.97	0.845	0.99	1	1	0	1.52
10	21	24.84	1.54	58.9	0.2382	0.96	0.8	0.8333	0.89	0.792	0.88	3	2	0	1.32
11	40	24.49	1.45	51.5	0.2117	1	0.79	0.7900	0.78	0.767	0.93	3	1	0	1.25
12	18	26.56	1.55	63.8	0.2212	0.99	0.91	0.9192	0.91	0.81	0.925	3	2	1	1.41
13	32	24.23	1.615	63.2	0.2457	0.995	0.86	0.8643	0.93	0.84	1	2	2	0	1.32
14	40	24.42	1.735	73.5	0.2492	1.04	0.945	0.9087	0.96	0.945	1.035	3	0	0	1.8
15	21	24.17	1.595	61.5	0.2408	0.96	0.772	0.8042	0.88	0.874	0.835	1	2	1	1.66
16	27	25.77	1.587	64.9	0.2411	1.065	0.83	0.7793	0.88	0.852	0.88	2	2	0	1.54

Note: A = age, B = BMI, C = body height, D = body weight, E = foot length with standing posture, F = hip circumference, G = waist circumference, H = waist-to-hip ratio, I = chest circumference, J = lower limb length, K = pelvis circumference, L = alignment of the heel, M = alignment of the shank, N = hallux valgus, WS = walking speed. In the heel and shank alignment, the deformity level is 0 = normal, 1 = mild, 2 = moderate, and 3 = severe. For the hallux valgus: 0 = normal, 1 = mild, 2 = severe. The deformity of the heel and shank includes inversion and eversion. Heel angle (degree) and shank angle: mild (< 4^◦^), moderate (4^◦^-8^◦^), and severe (> 8^◦^). Hallux angle (degree): normal (< 16^◦^), mild (16^◦^–30^◦^), severe (> 30^◦^)

**Table 4:** Kinematic parameters calculated by Anybody musculoskeletal model (N/kg)

No	HPF first peak (BW)	HPF second peak (BW)	KNF first peak (BW)	KNF second peak (BW)	PFF first peak (BW)	PFF second peak (BW)	AKF Peak (BW)	GRF first peak (BW)	GRF second peak (BW)
1	5.13	3.17	4.25	2.92	2.53	1.13	4.71	1.27	1.05
2	6.29	3.83	3.24	3.60	0.92	0.44	4.93	1.18	1.15
3	3.92	3.55	2.09	2.58	0.46	0.71	4.01	1.06	1.11
4	4.00	3.45	3.82	3.37	2.65	0.81	5.63	1.29	1.05
5	3.29	2.20	2.29	2.12	0.82	0.65	4.00	1.06	1.03
6	5.18	3.69	2.62	4.02	0.80	0.56	5.35	1.11	1.07
7	3.55	2.77	2.41	2.43	0.88	0.42	3.83	1.04	1.00
8	3.55	3.59	1.68	2.83	0.28	0.52	3.54	0.99	1.04
9	3.90	4.75	2.35	0.61	2.74	4.90	4.90	1.27	1.04
10	3.49	3.14	2.60	2.71	1.11	0.85	5.33	1.09	1.13
11	3.14	3.37	1.82	3.17	0.44	0.65	5.17	1.05	1.07
12	4.51	2.58	2.89	2.26	1.01	0.27	4.13	1.17	1.01
13	3.71	3.34	2.79	3.17	1.06	0.45	4.85	1.10	1.02
14	4.25	3.93	3.05	3.27	0.89	0.39	4.28	1.28	1.07
15	3.91	3.63	3.32	3.44	1.58	0.40	5.73	1.19	1.13
16	4.25	2.56	2.95	2.58	1.17	0.30	4.46	1.18	1.08

Abbreviation: *AKF*, ankle joint contact force; *GRF*, ground reaction force; *HPF*, hip joint contact force; *KNF*, knee joint contact force; *PFF*, patella-femur joint contact force
